# Pulsed Field Ablation: A Systematic Review

**DOI:** 10.1002/hsr2.70439

**Published:** 2025-05-19

**Authors:** Poe Nu Htay, Mya Hnin Aung, Thiri May Sin, May Thet Naing Oo, Sai Say Han, Eaint Nadi Naing, Myat Soe Thet, Khin Maung Htay

**Affiliations:** ^1^ King's College London, Faculty of Life Sciences and Medicine London UK; ^2^ University of Medicine 2 Yangon Myanmar; ^3^ Victoria Hospital Kirkcaldy UK; ^4^ University of Medicine Mandalay Myanmar; ^5^ Department of Obstetrics and Gynaecology Wrexham Maelor Hospital Wrexham UK; ^6^ Department of Surgery and Cancer Imperial College London London UK; ^7^ University of Medicine 1 Yangon Myanmar

**Keywords:** atrial fibrillation, cryoablation, pulsed‐field ablation, radiofrequency ablation, thermal ablation

## Abstract

**Background:**

Atrial fibrillation (AF) is the most prevalent cardiac arrhythmia, leading to significant health and economic burdens. Pulmonary vein isolation (PVI) is a key treatment strategy, with pulsed field ablation (PFA) emerging as a promising method due to its specificity and reduced collateral damage compared to traditional thermal ablation techniques like radiofrequency ablation (RFA) and cryoablation (CB).

**Materials and Methods:**

A comprehensive literature search was performed across multiple databases, including PubMed, Embase, and MEDLINE via Ovid, Scopus, Cochrane CENTRAL, and ClinicalTrials.Gov. Studies reporting on the efficacy and safety of PFA in AF treatment were selected and analyzed. Quality assessment of the studies was conducted using the Newcastle–Ottawa Scale.

**Result:**

Of the 440 articles initially identified, 28 met the inclusion criteria. PVI using PFA demonstrated high success rates, with most studies reporting over 90% success. Durability stands around 65% after 1 year. Mortality was 0.06%–0.32%, while stroke rate was 0.3%–4.4%. There were no reported oesophageal injuries or pulmonary vein stenosis due to the highly selective electroporation‐induced cell death caused by PFA rather than coagulative necrosis, sparing nearby structures. There is a short learning curve for PFA.

**Conclusion:**

PFA is a highly effective and safe ablation method. It offers an alternative to conventional thermal ablation strategies in the treatment of AF, showing promise to reduce the risk of collateral damage and complications associated with thermal ablation techniques. However, further research is needed to understand its long‐term efficacy and safety fully and to standardize procedural protocols for wider clinical application.

## 1 Introduction

Atrial fibrillation (AF), the most prevalent cardiac arrhythmia, presents a significant public health challenge, affecting not only patient morbidity and mortality but also contributing to substantial healthcare costs and utilization. This condition, characterized by an irregular and often rapid heart rate, significantly diminishes the quality of life and poses challenges in medical management [1, 2]. Among the various treatment options, pulmonary vein isolation (PVI) has emerged as a cornerstone strategy, particularly for symptomatic paroxysmal and persistent AF [3].

Traditionally, PVI has predominantly employed thermal energy sources such as radiofrequency ablation (RFA) and cryoablation (CB). Since its inception in 1998, catheter ablation using these modalities has focused on isolating arrhythmogenic foci within the pulmonary veins. However, these techniques have their limitations. Despite ongoing refinements in ablation strategies and energy delivery titration, thermal methods inherently risk collateral damage to cardiac and extracardiac tissues, including the oesophagus and bronchus [4, 5, 6, 7, 8, 9, 10, 11]. This collateral damage can lead to severe complications, such as pulmonary vein stenosis, phrenic nerve damage, and most alarmingly, atrio‐esophageal fistula, which carries a high mortality rate [12]. Furthermore, limiting energy delivery could result in decreased effectiveness in the treatment of AF.

In response to these challenges, pulsed field ablation (PFA) has emerged as an innovative technique. PFA represents a paradigm shift in the approach to AF ablation. By delivering high‐intensity electrical fields, PFA induces irreversible electroporation, effectively causing cell death with increased specificity and reduced collateral damage [13, 14, 15, 16, 17]. Reddy and colleagues first reported the clinical application of PFA in 15 patients with paroxysmal AF [18]. This method has shown promise in achieving high acute and long‐term PV isolation rates while maintaining a remarkably low complication profile. Notably, unlike thermal ablation methods, PFA has not been associated with collateral tissue damage, such as oesophageal lesions, a significant concern with traditional techniques [16, 17]. However, the full potential and limitations of PFA are still under investigation. Therefore, this systematic review aims to critically assess the existing literature on PFA, focusing on its efficacy, safety, and role in the broader context of AF management.

## 2 Materials and Methods

This systematic review follows the updated Preferred Reporting Items for Systematic Reviews and Meta‐Analyses (PRISMA) guidelines as revised in 2020 [19].

### 2.1 Literature Search

A comprehensive systematic literature search was performed to evaluate studies focused on recurrence and complications in patients with atrial fibrillation undergoing pulse field ablation or electroporation. The databases searched were PubMed, Medline via Ovid, Scopus, Embase, the Cochrane Central Register of Controlled Trials (CENTRAL), and ClinicalTrials.gov. Additionally, references from the selected studies and pertinent reviews were manually screened to identify any potentially relevant studies for inclusion.

### 2.2 Search Strategy

The studies included in this review were not restricted by their year of publication. The literature search was conducted in October 2024. Keywords used for this literature search were combined using Boolean operators: “(Atrial fibrillation) AND ((pulsed‐field ablation) OR (electroporation)) AND ((Recurrence) OR (complications)).”

### 2.3 Study Selection

This review included original research studies that reported on success, recurrence and complications in patients with atrial fibrillation treated through pulse field ablation or electroporation. Non‐English studies, animal studies, reviews, case reports, and case series were excluded from this review.

### 2.4 Data Extraction and Outcome Measures

The included studies were independently reviewed and data was extracted by two authors. Any disagreements that arose were resolved through consensus with a third author. Data extracted included (i) study characteristics such as the aim, design, and period of the study, (ii) a description of the patient population, including inclusion and exclusion criteria; (iii) characteristics of the patient population, encompassing age, gender, comorbidities, and medication treatment, and (iv) details of the pulse field ablation procedure, including duration and outcomes (success rate, complications), and where applicable, comparisons with other methods.

### 2.5 Quality Assessment

Two authors independently evaluated the quality of the included studies utilising the Newcastle–Ottawa Scale (NOS) for cohort studies [20]. Studies with scores ranging from 7 to 9 were classified as “good quality.” Those obtaining scores between 4 and 6 were deemed “fair quality,” while studies that achieved scores from 0 to 3 were categorized as “poor quality.”

## 3 Results

**Figure 1:**
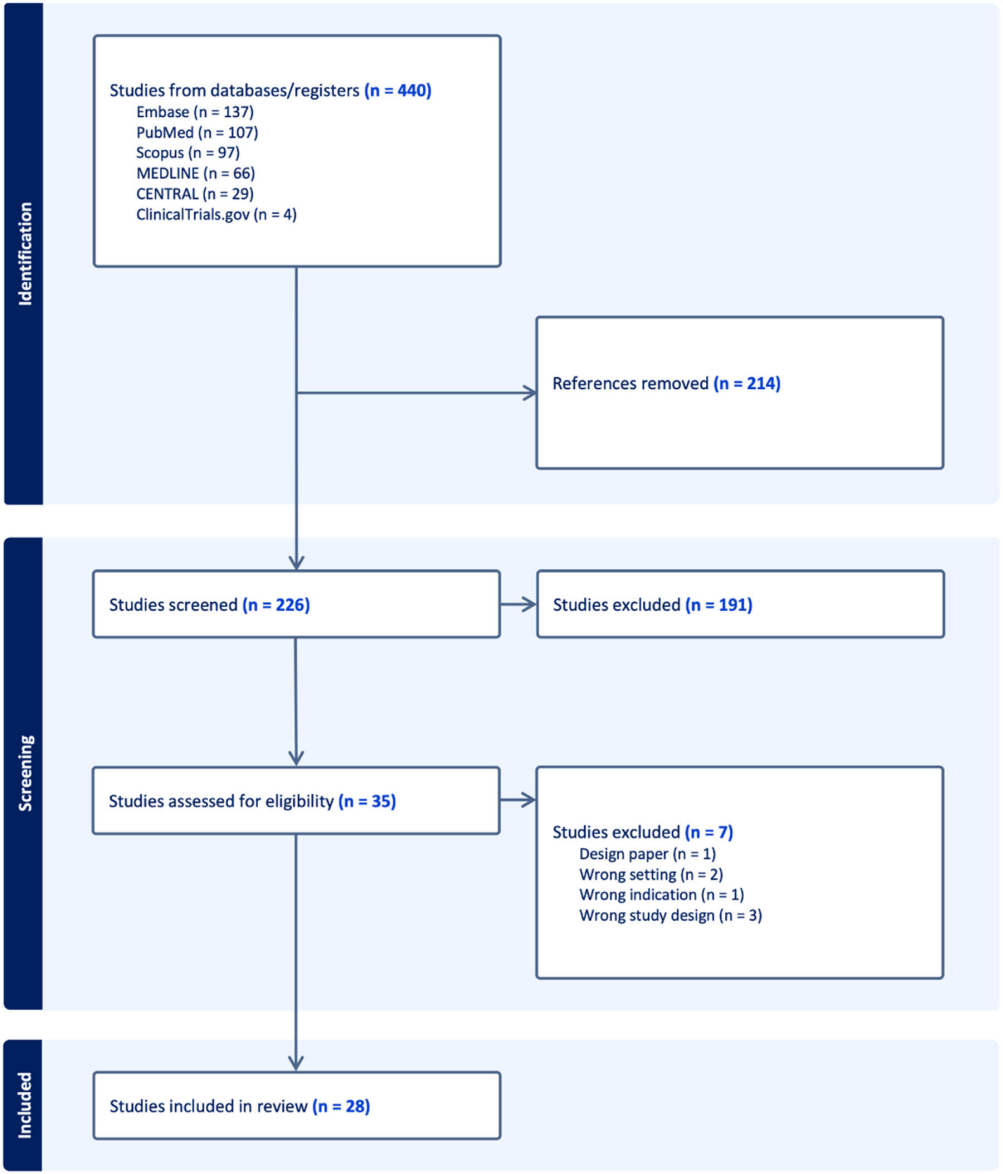
PRISMA flowchart of the included studies.

A total of 440 research articles were initially identified from the database. Subsequent to excluding 214 studies as duplicates, 226 studies underwent preliminary title and abstract screening to determine their eligibility for inclusion. This was followed by a detailed full‐text evaluation of 35 studies, ultimately resulting in the inclusion of 28 studies in the systematic review, as described in Figure 1. Study characteristics are described in Table 1. The quality assessment of cohort studies using the NOS showed eight good quality studies, and the quality of the remaining studies was fair (Table S1). The risk of bias assessment of the randomized controlled trial was described separately in Table S2.

**Table 1:** Characteristics of the studies included.

Author	Year	Design	Type of catheter	PFA patient no.	Comparison with other ablations	Reported outcomes
Blockhaus et al.	2023	R	Pentaspline	23	CB (*n* = 20)	– 100% success rate for PFA, 100% for CB – 0% early reconnections – 4.34% stroke, no other complications
Cochet et al.	2021	P	Pentaspline	18	RFA (*n* = 16), CB (*n* = 7)	– 100% success rate for PFA, RFA, CB – Thermal ablations causes 43% injury to oesophagus and aorta – No oesophageal injury in PFA, but 33% aortic injury in PFA – CMR 3 months postprocedure shows complete resolution
Ekanem et al.	2022	R, M	Pentaspline	1758	N/A	– 99.9% success rate – 0.97% pericardial tamponade – 0.4% stroke – 0.06% mortality
Füting et al.	2023	P	Pentaspline	60	N/A	– 100% success rate – No bronchial lesions or ulcers postprocedure bronchoscopy – No complications
Füting et al.	2022	P	Pentaspline	30	N/A	– 100% success rate – No oesophageal or phrenic nerve injury – 3.3% pericardial tamponade
Gunawardene et al.	2022	P	Pentaspline	20	N/A	– 100% success rate – 6.25% acute PV reconnection – 5% coronary spasm
Guo et al.	2023	P	Circular	18	N/A	– 100% success rate – No change in serum nerve injury markers pre and postablation – No complications
Kawamura et al.	2021	R, M	Pentaspline	20	RFA (*n* = 29), CB (*n* = 6), VGLB (*n* = 4)	– No significant different of PV isolation areas in all groups – Notch‐like normal voltage areas in posterior wall in CB, VGLB, but not in PFA and RFA groups
Kuroki et al.	2020	P	Pentaspline	37	RFA (*n* = 43)	– PV ostial diameter decrease more with RFA compared to PFA – RFA group: 9.0% mild PV stenosis (30%–49%), 1.8% moderate PV stenosis (50%–69%), 1.2% severe PV stenosis (70%–100%) – PFA group: 0% PV stenosis
Lemoine et al.	2022	P, M	Pentaspline	138	N/A	– 100% success rate – 6% repeated PFA applications due to acute reconnections – 0.7% pericardial tamponade, 0.7% transient ST elevation, 2.2% groin haeatoma – 90% freedom from arrhythmia in paroxysmal AF, 60% in persistent AF
Loh et al.	2020	P	Circular	10	N/A	– 100% success rate – No acute PV reconnections during waiting period and adenosine test – No complications
Magni et al.	2022	P	Pentaspline	100	N/A	– 100% success rate – Mean application per PV = 8.1 ± 0.6 – 2% bleeding at access site
Magni et al.	2023	R, M	Pentaspline	14	N/A	– 14 redo‐PFA procedures – 85.7% recurrence of AF, remaining had atrial flutter or tachycardia – 35.7% had no PV reconnection, 21.4% had one PV reconnection, 14.3% had two PV reconnections, 28.6% had three PV reconnections
Reddy et al.	2020	P, M	Lattice‐tip	36	RFA + PFA (*n* = 40)	– 100% success rate in all groups – 5.6% mucosal oesophageal injury in RFA + PFA group, 0% in PFA group
Reddy et al.	2021	P, M	Single shot—type multielectrode PFA Catheter, Deflectable focal PFA catheter	Monophasic (*n* = 15), Biphasic optimized (*n* = 49), Biphasic other (*n* = 57)	N/A	– 100% success rate in all groups – PVI durability 84% with biphasic optimized PFA, 58% with biphasic other and 18% with monophasic PFA
Reddy et al.	2018	P, M	Pentaspline	Endocardial (*n* = 15), Epicardial (*n* = 7)	N/A	– 100% success rate for endocardial PFA – 86% success rate for epicardial PFA due to technical limitations – No complications
Reddy et al.	2023	RCT, M	Pentaspline	305	RFA (*n* = 167), CB (*n* = 135)	– 73.3% success rate in PFA, 71.3% in thermal ablation (RFA + CB) at 1 year – Mortality: 0.3% in PFA, 0% in thermal ablation – Stroke: 0% in PFA, 0.3% in thermal ablation – Pericardial tamponade: 0.7% in PFA, 0% in thermal ablation
Ruwald et al.	2023	P	Pentaspline	121	N/A	– 98% success rate – 0.8% phrenic nerve injury – 18.2% clinically significant recurrence – 6.6% required redo‐ablation
Ruwald et al.	2023	P	NA	51	N/A	– 94% success rate – First pass was successful in 97% of all PVs, 100% for both cavotricuspid isthmus and mitral isthmus, 82% for left atrial posterior wall – 2% ST elevation, resolved with intravenous nitroglycerine – 2% heart block requiring temporary pacing more than 24 h
Schmidt et al.	2022	P	Pentaspline	191	N/A	– 100% success rate, 99.5% success rate with first pass – 1% stroke – 19% silent cerebral ischaemia – 9% recurrence of atrial tachyarrhythmia
Schmidt et al.	2023	R, M	Pentaspline	1233	N/A	– 99.96% success rate – 1 patient died from peri‐procedural stroke – 1.1% pericardial tamponade – 0.41% stroke – 0.16% TIA – 1.9% minor complications
Sohns et al.	2023	P	Pentaspline	10	N/A	– 100% success rate – 90% freedom from recurrence at 7 months – No oesophageal injury or PV stenosis
Tohoku et al.	2023	P	Pentaspline	25	N/A	– 25 patients for redoablation – 64% atrial tachycardia, 24% AF, 1 atrial flutter – 9.1% PV reconnection
Turagam et al.	2023	P	Spherical	21	N/A	– 100% success rate – PVI durability 62.5% with one PFA application, 100% with three PFA applications – No oesophageal injury on OGD
Turagam et al.	2023	R, M	Pentaspline	1568	N/A	– 99.2% success rate – 1.9% overall major adverse event rate – 1.1% pericardial tamponade – 0.4% stroke – 0.06% mortality
Urbanek et al.	2023	R	Pentaspline	200	CB (*n* = 200)	– 100% success rate in PFA, 98% in CB group – Median procedure time was significantly shorter in PFA than CB (34.5 vs. 50 min) – Overall complication rate 3.0% in PFA, 6.5% in CB
Verma et al.	2023	P, M	Circular	383	N/A	– PFA PVI durable at 66.2% for paroxysmal AF and 55.1% for persistent AF at 1 year – 0.7% stroke – 0.7% pericardial tamponade
Verma et al.	2023	P, M	Circular	300	N/A	– No arrhythmia burden in 69.4% of paroxysmal AF, 62.2% of persistent AF – Less than 10% of arrhythmia burden in paroxysmal AF patients had quality of life improvement – Repeat ablations and cardioversion increase with higher arrhythmia burden

Abbreviations: AF, atrial fibrillation; CB, cryoablation; M, multicentre study; OGD, oesophagogastroduodenoscopy; P, prospective cohort; PFA, pulsed‐field ablation; PV, pulmonary vein; R, retrospective cohort; RCT, randomized controlled trials; RFA, radiofrequency ablation; VGLB, visually guided laser balloon.

### 3.1 Pulmonary Vein Isolation Success

Out of 28 studies, 22 studies reported the PVI success rate. Remarkably, 16 of these studies reported a 100% success rate of PVI using PFA [15, 16, 21, 22, 23, 24, 25, 26, 27, 28, 29, 30, 31, 32, 33, 34]. A comprehensive multicentre survey, which included 24 centers and encompassed 1758 patients, exhibited an exceptionally high success rate of 99.9% for PVI [35]. The EU‐PORIA registry involving 1233 patients demonstrated a PVI success rate of 99.96%, comparable to other studies [36]. At 1 year, 72% of pulmonary veins remained durably isolated. In a comparative study by Reddy and colleagues, the success rates of endocardial and epicardial PVI using PFA were evaluated, yielding success rates of 100% and 85.7%, respectively [18]. The relatively lower success rate of epicardial PFA, which was used in patients undergoing cardiac surgery, was attributed to technical complexities encountered during its application. Furthermore, despite a diverse group of 77 operators, as reported in a study by Turagam and colleagues, demonstrated a high success rate of 99.2% in achieving PVI using PFA [37]. A similar result was also achieved in a randomized controlled trial (ADVENT), which included 305 PFA patients, with a success rate of 99.6%, which stood at 73.4% at 1 year [38].

### 3.2 Durability of PFA and Recurrence of Atrial Arrhythmia

The durability of PVI with PFA varied significantly based on the energy modalities employed. The efficacy of a single PFA application stood at 62.5%, it was observed that this rate could be enhanced to 100% with the implementation of three PFA applications [32]. During systematic remapping of all patients, acute PV reconnections were observed in approximately 2%–20% of cases [26, 29, 38]. Tendency for reconnections occurs predominantly around the superior pulmonary veins [15].

Three months post the index procedure, the durability of PVI was 64.5% [15]. The ADVENT randomized controlled trial reported a durable PFA success rate of 64.8% at 1 year [38]. A significant number of studies noted an early recurrence of atrial arrhythmias, with rates ranging between 2% and 20% within the same 3‐month period [25, 27, 28, 30, 33, 39]. This recurrence rate showed comparability to that observed in CB procedures, where the PFA group exhibited a 19.5% recurrence rate against a 21.5% rate in the CB cohort at 3 months [33]. Some studies reported a symptom‐free rate of 80%–90% at 3 months [21, 34], with one specific study reporting a 90% symptom‐free rate at 7 months [31]. In the EU‐PORIA registry, freedom from arrhythmia was 74% at 1 year. Paroxysmal AF patients showed higher success rates at 80% compared to persistent AF patients at 66% [36]. The average timeframe for recurrence post‐PFA was around 5 months [40]. The majority of patients experiencing recurrence presented with AF, with recurrence rates ranging between 86% and 89%, while the remainder suffered from atrial flutter and other forms of atrial tachycardia [39, 40]. Between 2% and 10% of patients required a redo procedure [22, 30, 31, 33, 39, 41]. Redo procedures typically had a higher PV reconnection rate of 35.8% during remapping [40]. The left superior pulmonary vein was identified as the most common reconnection site [41].

### 3.3 Antral Lesion

Uniform alterations in the antral tissue of the pulmonary vein were observed through MRI 3 months after PVI with PFA, without any collateral tissue damage [31]. The first pass attempt at achieving acute isolation was efficacious in 97% of PVI instances, achieved complete success in both the cavotricuspid isthmus and mitral isthmus block, and was effective in 82% of cases for the left atrial posterior wall, thereby establishing a bidirectional electrophysiological block [42]. The isolated surface area ratio post PVI with PFA was recorded at 67.03 ± 12.69%, a figure significantly greater than the 57.39 ± 10.91% noted when using the CB technique [23]. On the other hand, Kawamura and colleagues found no significant variance in the isolated surface areas when comparing the PFA (11.0 ± 3.4 cm^2^) and RFA cohorts (10.6 ± 3.4 cm^2^) [43].

### 3.4 Mortality and Complications

The mortality rate associated with PVI using PFA was 0.06%–0.32% [35, 36, 38]. Stroke incidence postprocedure varied between 0.3% and 4.3% [23, 30, 34, 35, 36, 39], while transient ischemic attacks (TIA) were observed in 0.1%–0.8% of cases [15, 36, 37]. Silent cerebral ischemia, detectable on radiological imaging but without clinical symptoms, occurred in 8.9% to as high as 18.8% of patients [29, 32, 34]. Pericardial effusion leading to cardiac tamponade requiring evacuation occurred in 0.3%–1.6% [15, 21, 26, 30, 33, 34, 35, 36, 39]. Transient bradyarrhythmia, including asystole, was the most common complication in about 33% of the patients [21]; however, a temporary pacemaker was required in only 1.9% of the patients with new heart block after PFA [43]. Notably, no oesophageal injury was observed in the PFA patients [16, 29, 38]. The oesophageal injury was observed in 5.6% when RFA was used in conjunction with PFA [29], while the injury was observed in up to 43% of RFA and CB groups [16].

Pulmonary stenosis was absent in all PFA patients; in contrast, mild to moderate pulmonary vein stenosis occurred in 32.5% of RFA patients [42]. Incidences of phrenic nerve injury ranged from 0.3% to 1.0% [30, 33, 35, 36, 37, 39]. Aortic lesions were noted in 33% of PFA and 43% of RFA patients, with complete resolution in follow‐up MRI imaging at 3 months [16]. Other rare complications included coronary artery spasm (0.06%–5.0%) [24, 35, 36, 37], short‐term cough (7%) [22], persistent cough and haemoptysis beyond 6 weeks (0.06%–0.08%) [35, 36], Dressler's syndrome (0.16%–0.27%) [36, 41], groin pseudoaneurysm (2.5%) [33], and groin haematoma necessitating evacuation (1.3%) [29].

### 3.5 Learning Curve

There is a short learning curve for the PFA [26, 28, 36, 39]. Comparison between the initial and latter halves of the patient cohort treated by four operators revealed a significant decrease on average fluoroscopy time, with no change in the average procedure duration. However, a substantial reduction was observed in both procedure (85 ± 34 min to 72 ± 18 min) and fluoroscopy times (22 ± 9 min to 16 ± 4 min) when the initial 10 and final 10 cases were compared. The standard deviation for procedure time stabilized at 16 min after 15 procedures [39]. The learning curve for RFA and CB was relatively longer when compared to that of PFA [28].

## 4 Discussion

PV reconnections for thermal ablation strategies (RFA, CB) were reported between 22% and 38% [44, 45, 46, 47, 48], with some studies as high as 50%–62.5% [49, 50]. This is probably due to the failure of complete transmural lesion formations without gaps. Biphasic optimized PFA has lower rates of 2%–20% of acute PV reconnections on systematic remapping postablation [26, 29, 38]. This can be attributed to the nature of PFA, causing highly selective transmural homogenous lesions without collateral damage. On the other hand, RF ablations could result in disorganized heterogenous lesions [51]. Although it was previously shown adequate lesions can be formed even if there is a gap up to 2 mm between the catheter and the atrium [52], the use of intracardiac echocardiography can improve the efficacy of PFA by enhancing the contact surface between the catheter and myocardium [15]. However, the PV reconnection rate for PFA was 35.8% during the redo procedures remapping [40]. This is almost twice the rate found on systematic remapping postablation because all patients who come back for redo procedures have atrial tachyarrhythmias and are expected to have a higher rate of PV reconnection. Many studies report symptom‐free rates of 80%–90% during the initial 3‐month period [21, 34].

In the context of thermal ablation strategies (RFA and CB), rates of PV reconnections have been found in the range of 22%–38% [44, 45, 46, 47, 48], with certain studies indicating rates as high as 50%–62.5% [49, 50]. This phenomenon is likely attributable to the failure to form complete transmural lesions that are devoid of gaps. In contrast, Biphasic Optimized PFA demonstrates a markedly lower incidence of acute PV reconnections during systematic remapping, reported to be between 2% and 20% [26, 29, 38]. This reduced rate can be ascribed to the distinctive nature of PFA in generating highly selective and homogeneous transmural lesions, which importantly occur without causing collateral damage. Conversely, RF ablations are prone to creating disorganized and heterogeneous lesions [51]. Previous research has demonstrated that adequate lesion formation is feasible even with a gap of up to 2 mm between the catheter and the atrium [52]; however, the application of intracardiac echocardiography has been shown to augment the effectiveness of PFA by improving the contact interface between the catheter and myocardium [15]. The PV reconnection rate for PFA was recorded at 35.8% during redo procedures involving remapping [40]. Numerous studies have reported symptom‐free rates of 80%–90% during the initial 3‐month postablation period [21, 34].

PFA exhibits a commendably safe profile, with an overall mortality rate ranging from 0.06% to 0.32% [35, 36, 38], a figure that aligns closely with the 0.6% mortality rate observed in thermal ablation techniques across a cohort of over 100,000 patients [53]. The incidence of stroke in PFA cases is at 0.3%–4.3% [23, 30, 34, 35, 36, 39], paralleling the 3% rate associated with thermal ablations [53]. Notably, silent cerebral ischemic events, which are clinically asymptomatic, occur in approximately 9% of PFA patients [29], with literature indicating a broader range of 2%–40% for such events for other various ablation modalities [4, 54]. A distinctive advantage of PFA lies in its ability to target myocardial tissue selectively, sparing adjacent structures like the oesophagus. Remarkably, oesophagal injuries have not been reported in PFA studies, even in the absence of specific oesophagal protection protocols, potentially removing the need for routine esophagogastroduodenoscopy [15, 21]. This sparing effect is attributed to the reliance of PFA on electroporation, which induces pore formation rather than coagulative necrosis of matrix tissues [18]. However, the descending aortic tissues are found to be sensitive to electroporation, and the aortic lesions have been observed in up to 43% of PFA patients, though these effects typically resolve within a few months without further complications [16]. To date, there have been no reports of oesophagal fistula formation associated with PFA. Additionally, PFA does not result in pulmonary vein (PV) stenosis, a contrast to thermal ablation strategies, which have been linked to PV stenosis rates of 31.4% for mild stenosis (30%–49%), 4.3% for moderate stenosis (50%–69%), and 0.7% for severe stenosis (70%–100%) [42]. There have been no incidences of PV stenosis caused by PFA reported thus far.

This systematic review, while comprehensive in its assessment of clinical aspects of PFA for atrial fibrillation, is not without limitations. The variability in study designs and methodologies may introduce bias and limit the generalizability of the findings. One of the major limitations is that several studies used different types of catheters. They come in various shapes, protocols, and mapping techniques, so the clinical outcomes of one type of catheter may not be applicable to other types used in different studies. We also note that there is only one randomized controlled trial so far. The predominance of short to medium‐term data limits insights into the long‐term efficacy and safety of PFA. Differences in PFA application techniques and procedural protocols across studies could sometimes lead to inconsistent outcomes and the lack of extensive comparative analyses with other ablation techniques, such as RFA and CB. Future research addressing these gaps is essential for a better understanding of PFA's role in managing atrial fibrillation.

## 5 Conclusion

PFA stands out as a highly effective and safe method for ablation, presenting a viable alternative to traditional thermal ablation strategies in the management of AF. Its potential to minimize collateral damage and complications, commonly seen with thermal ablation techniques, is promising. Nonetheless, further research is essential to comprehensively understand its long‐term effectiveness and safety, as well as to establish standardized procedural protocols for broader clinical use.

## Author Contributions

**Poe Nu Htay:** conceptualization, data curation, formal analysis, investigation, methodology, project administration, resources, validation, writing – review and editing, writing – original draft. **Mya Hnin Aung:** conceptualization, data curation, formal analysis, investigation, methodology, project administration, resources, validation, writing – original draft, writing – review and editing. **Thiri May Sin:** conceptualization, data curation, formal analysis, investigation, methodology, writing – original draft. **May Thet Naing Oo:** conceptualization, data curation, formal analysis, investigation, methodology, writing – original draft. **Sai Say Han:** conceptualization, investigation, writing – original draft, methodology, formal analysis, data curation. **Eaint Nadi Naing:** conceptualization, investigation, writing – original draft, methodology, formal analysis, data curation. **Myat Soe Thet:** conceptualization, data curation, formal analysis, investigation; methodology, project administration, resources, supervision, validation, writing – original draft, writing – review and editing. **Khin Maung Htay:** conceptualization, project administration, resources, supervision, validation, writing – original draft, writing – review and editing.

## Conflicts of Interest

The authors declare no conflicts of interest.

## Transparency Statement

The lead author Myat Soe Thet affirms that this manuscript is an honest, accurate, and transparent account of the study being reported; that no important aspects of the study have been omitted; and that any discrepancies from the study as planned (and, if relevant, registered) have been explained.

## Supporting information

Supporting information.

## Data Availability

All studies included in this systematic review are publicly available on PubMed. Any additional data generated or analyzed during this study are included within the article and its Supporting Information. Further details can be obtained from the corresponding author upon reasonable request.
