# Supplementary material for: Pulsed Field Ablation: A Systematic Review
Source: Health Sci Rep. 2025 May 19;8(5):e70439. doi: 10.1002/hsr2.70439 (PMC12086654; doi:10.1002/hsr2.70439)
Supplement: Supplementary file 1 — Supporting information. [file HSR2-8-e70439-s001.docx]

## SUPPLEMENTARY MATERIALS

Supplementary Table 1: Quality assessment of the included studies using the Newcastle-Ottawa Scale

| **Study ID** | **Selection of Study Groups**  **(0-4)** | | | | **Comparability**  **(0-2)** | **Assessment of Outcome**  **(0-3)** | | | **Total Score**  **(0-9)** |
| --- | --- | --- | --- | --- | --- | --- | --- | --- | --- |
|  | **Representativeness of the exposed cohort** | **Selection of the non-exposed cohort** | **Ascertainment of exposure** | **Demonstration of the outcome is not present at the beginning** | **Comparability of cohorts on the basis of the design or analysis** | **Assessment of outcome** | **Enough follow-up length for outcomes to occur** | **Adequacy of follow-up of cohorts** |  |
| Blockhaus 2023 | 1 | 1 | 1 | 0 | 2 | 1 | 1 | 1 | 8 |
| Cochet 2021 | 1 | 1 | 1 | 0 | 2 | 1 | 1 | 1 | 8 |
| Ekanem 2022 | 1 | 0 | 1 | 0 | 0 | 1 | 1 | 1 | 5 |
| Füting 2023 | 1 | 1 | 1 | 0 | 2 | 1 | 1 | 1 | 8 |
| Füting 2022 | 1 | 0 | 1 | 0 | 0 | 1 | 1 | 1 | 5 |
| Gunawardene 2022 | 1 | 0 | 1 | 0 | 0 | 1 | 1 | 1 | 5 |
| Guo 2023 | 1 | 0 | 1 | 0 | 0 | 1 | 1 | 1 | 5 |
| Kawamura 2021 | 0 | 0 | 1 | 0 | 2 | 1 | 1 | 1 | 6 |
| Kuroki 2020 | 1 | 1 | 1 | 0 | 2 | 1 | 1 | 1 | 8 |
| Lemoine 2022 | 1 | 0 | 1 | 0 | 0 | 1 | 1 | 1 | 5 |
| Loh 2020 | 1 | 0 | 1 | 0 | 0 | 1 | 1 | 1 | 5 |
| Magni 2022 | 1 | 0 | 1 | 0 | 0 | 1 | 1 | 1 | 5 |
| Magni 2023 | 1 | 0 | 1 | 0 | 0 | 1 | 1 | 1 | 5 |
| Reddy 2020 | 0 | 1 | 1 | 0 | 2 | 1 | 1 | 1 | 7 |
| Reddy 2021 | 0 | 1 | 1 | 0 | 2 | 1 | 1 | 1 | 7 |
| Reddy 2018 | 0 | 1 | 1 | 0 | 2 | 1 | 1 | 1 | 7 |
| Ruwald 2023 -a | 1 | 0 | 1 | 0 | 0 | 1 | 1 | 1 | 5 |
| Ruwald 2023 - b | 1 | 0 | 1 | 0 | 0 | 1 | 1 | 1 | 5 |
| Schmidt 2022 | 0 | 0 | 1 | 0 | 0 | 1 | 1 | 1 | 4 |
| Schmidt 2023 | 1 | 0 | 1 | 0 | 0 | 1 | 1 | 1 | 5 |
| Sohns 2023 | 1 | 0 | 1 | 0 | 0 | 1 | 1 | 1 | 5 |
| Tohoku 2023 | 1 | 0 | 1 | 0 | 0 | 1 | 1 | 1 | 5 |
| Turagam 2023 - a | 1 | 0 | 1 | 0 | 0 | 1 | 1 | 1 | 5 |
| Turagam 2023 - b | 1 | 0 | 1 | 0 | 0 | 1 | 1 | 1 | 5 |
| Urbanek 2023 | 1 | 1 | 1 | 0 | 2 | 1 | 1 | 1 | 8 |
| Verma 2023 - a | 1 | 0 | 1 | 0 | 0 | 1 | 1 | 1 | 5 |
| Verma 2023 - a | 0 | 0 | 1 | 0 | 0 | 1 | 1 | 1 | 4 |

Good quality (7-9 score), Fair quality (4-6 score), Poor quality (0-3 score)

Supplementary Table 2: Cochrane Risk of Bias Assessment for Randomised Controlled Trials (RCTs)

| **Study** | **Random sequence generation** | **Allocation concealment** | **Blinding of participants** | **Blinding of outcome assessment** | **Incomplete outcome data** | **Selective reporting** | **Other bias** |
| --- | --- | --- | --- | --- | --- | --- | --- |
| Reddy et al. 2023 | **(+)** | **(-)** | **(+)** | **(?)** | **(+)** | **(+)** | **(+)** |

(+) = Low risk of bias, (-) = High risk of bias, (?) = Unclear risk of bias
